# Demonstrating an Adult Ventricular Septal Defect Using Non-obstructive General Angioscopy

**DOI:** 10.7759/cureus.37673

**Published:** 2023-04-17

**Authors:** Sei Komatsu, Satoru Takahashi, Mitsuhiko Takewa, Tomoki Ohara, Chikao Yutani, Kazuhisa Kodama

**Affiliations:** 1 Cardiovascular Center, Osaka Gyoumeikan Hospital, Osaka, JPN

**Keywords:** cardiology, imaging, non-obstructive general angioscopy, congenital heart disease, ventricular septal defect

## Abstract

A ventricular septal defect (VSD) is a common congenital heart disease, and the transcatheter technique for VSD requires practical guidance because it requires a complicated procedure. A non-obstructive angioscopy catheter system via the right ventricle successfully revealed an approximately 3-mm VSD with the shape of a rugby ball at the center of the white membranous septum of Kirklin type II in an older female with suspected coronary artery disease. A white membranous terraced septum was observed to be surrounded by a reddish ventricle. Conservative therapy was performed for her VSD because she did not meet the criteria for surgical treatment.

## Introduction

Ventricular septal defect (VSD) ranks among the most prevalent types of congenital heart disease [1]. About 40% of congenital heart abnormalities are represented by VSDs [2]. Natural history depends on the characteristics of the anomaly, such as the size and anatomical associations of the anomaly, and the patient’s age [2]. A part of VSDs spontaneously closes [3]. The primary treatment for VSD is surgery [4], but catheter-based treatment has recently begun to emerge [5]. Two-dimensional echocardiography and three-dimensional imaging by echocardiography, magnetic resonance imaging, and computed tomography are used to evaluate the characteristics, such as the type, size, and number of defects, besides cardiac catheterization [6]. Non-obstructive general angioscopy (NOGA) can be observed inside any vessel [7] and aortic valve [8]. NOGA is a 6-Fr system and can be potentially used for the intervention. We reported a patient with a VSD observed by NOGA.

## Case presentation

**Figure 1:**
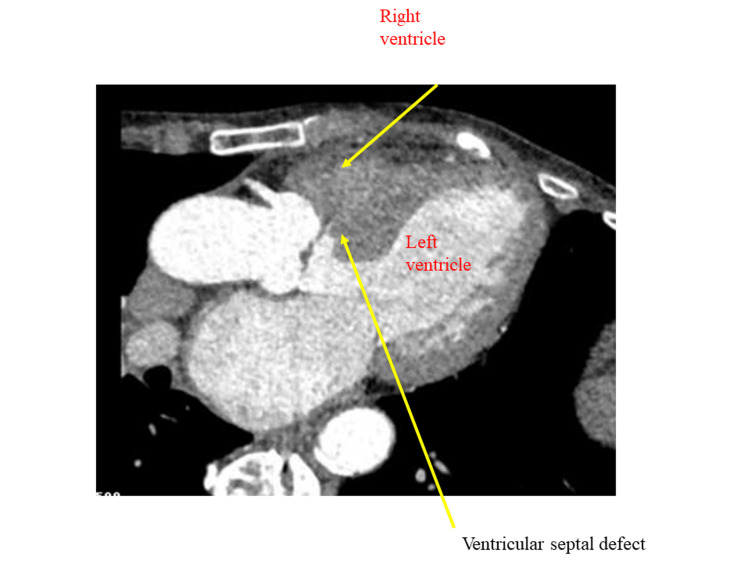
Four-chamber view of computed tomography angiography of the heart. An arrow shows contrast media from the left ventricle to the right ventricle through the ventricular septal defect below the aortic valve.

**Figure 2:**
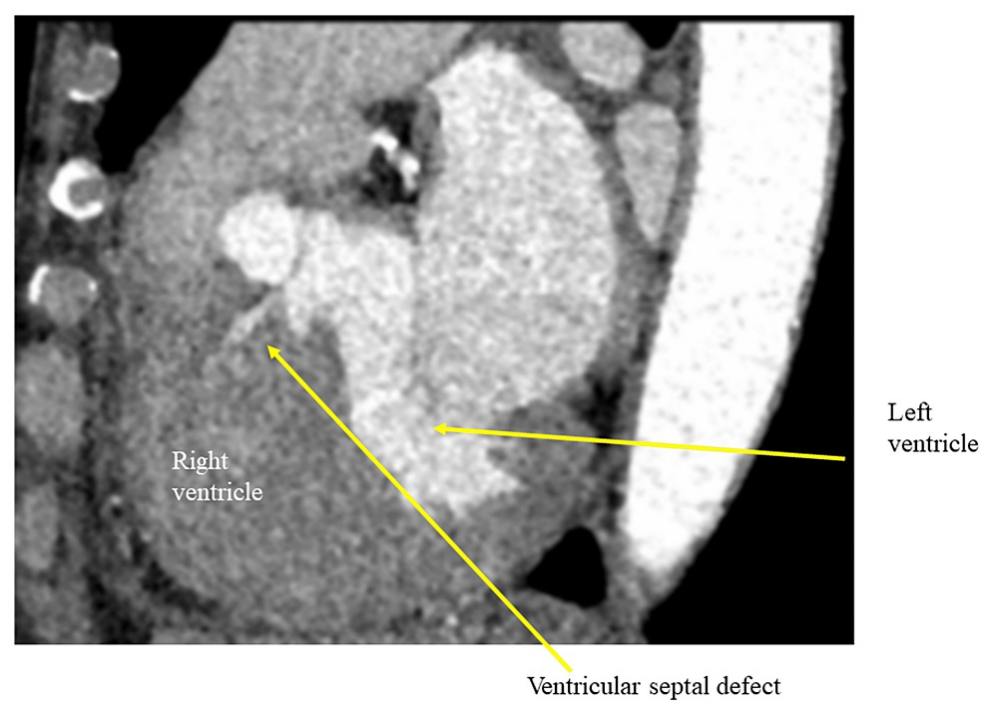
Sagittal view of computed tomography angiography of the heart. Contrast media from the left ventricle to the right ventricle through the ventricular septal defect is shown below the aortic valve by an arrow.

A 73-year-old female complaining of atypical chest pain and dyspnea on effort was admitted to our hospital. She had dyslipidemia and hyperthyroidism and was also on medication. A holosystolic murmur III/VI along the lower left sternal border was heard upon auscultation. Echocardiography and computed tomography angiography using dual-source computed tomography (Definition Flash, Forchheim, Germany) revealed a Kirklin type II VSD (Figure 1 and Figure 2).

**Figure 3:**
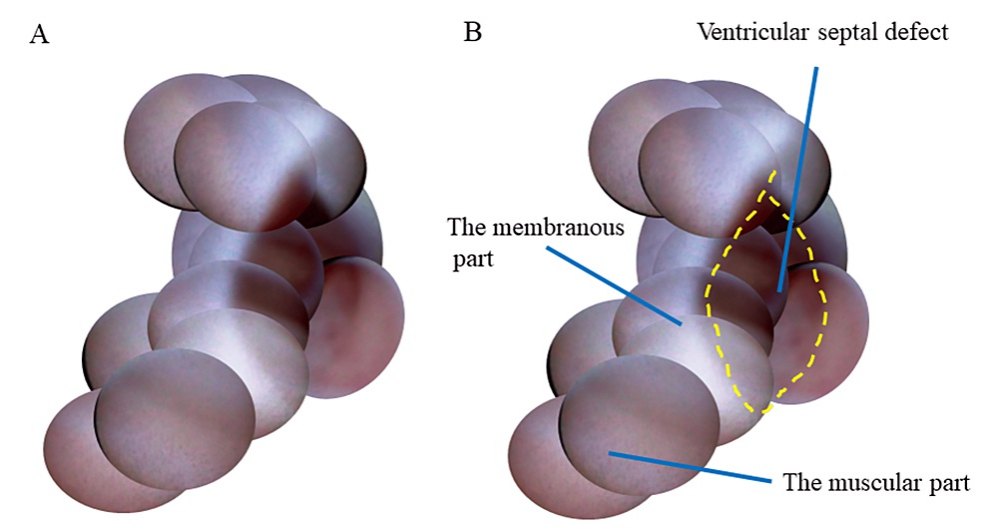
Angioscopic images of the ventricular septal defect without (A) and with (B) caption. The ventricular septal defect surrounded by the membranous part and muscular part of the right ventricle was observed.

The diameter of the defect was 3.1 mm, while the Qp/Qs ratio was 1.07. Because coronary artery disease was suspected in the left circumflex coronary artery from the coronary computed tomography angiography, cardiac catheterization was performed. Coronary angiography revealed a moderate coronary stenosis in the middle of the left circumflex coronary artery. A pressure study with the Swan-Ganz catheter showed that the pulmonary artery pressure was 24/10 mmHg. NOGA (Inter-tec Medicals Co., Ltd, Osaka, Japan) [7] with a 6-Fr Ikari Left catheter system via the right femoral vein was performed to demonstrate the VSD. A white membranous terraced septum was observed to be surrounded by a reddish ventricle. An approximately 3-mm VSD with the shape of a rugby ball at the center of the white membranous septum was detected (Figure 3 and Video 1).

**Video 1:** Angioscopic video of the ventricular septal defect.

The patient’s symptoms were not thought to be related to coronary heart disease or congenital heart disease, and she received no intervention.

## Discussion

The estimated incidence of VSD is between 5 and 50 cases per 1,000 live births [9]. The most common types are perimembranous VSDs, followed by muscular, inlet/atrioventricular canal-type, and outlet/conal septal defects [10]. Our patient’s VSD was of perimembranous type. NOGA clearly demonstrated the membranous part and the muscular part. Lack of pulmonary hypertension because of a small shunt might bring a good prognosis for the patient. Surgery for patients with perimembranous VSD is safe and effective at a young age [6]. The technique and device for transcatheter VSD have developed consistently [11,12]. The transcatheter technique is slightly complicated to perform and concerns about conduction problems, especially after membranous VSD closure with devices remain [6,12]. Fluoroscopy and echocardiographic guidance are used [10]. NOGA is an invasive device to demonstrate vessels [6] and inside heart chambers [7]. Recently, the endothelialization on the atrial septal occluder device [13] observed by NOGA was reported. When exploring the VSD by NOGA, it was found that the structure was more complex than the images observed by computed tomography. The three-dimensional structure of the terraced septum to a defect might make catheter intervention difficult. NOGA is used as a guide for other kinds of interventions. NOGA-assisted thoracic endovascular repair was helpful in determining the precise position of a graft by revealing abnormal conditions [14]. NOGA might provide additional unique information in the preoperative assessment of VSD; hence, accumulating case studies in which NOGA is used is necessary.

## Conclusions

A patient with an adult VSD of Kirklin type II was evaluated by NOGA, besides suspected coronary artery disease. NOGA clearly demonstrated the membranous part and the muscular part. NOGA may be used for guiding or evaluating an intervention for structural heart disease. NOGA may also be helpful for the preoperative assessment of VSD.
